# Efficacy of inhaled tobramycin in severe nosocomial pneumonia

**DOI:** 10.1186/cc10678

**Published:** 2012-03-20

**Authors:** A Kuzovlev, S Polovnikov, V Stec, V Varvarin

**Affiliations:** 1 V.A. Negovsky Scientific Research Institute of General Reanimatology RAMS, Moscow, Russia; 2 N.N. Burdenko Main Clinical Military Hospital, Moscow, Russia

## Introduction

Nosocomial pneumonia (NP) is one of the most prevalent complications in ICUs. The efficacy of inhaled antibiotics in treatment of NP was shown in several research works. The aim of this study was to estimate the efficacy of inhaled tobramycin (IT) as an adjunct to systemic antibiotics in the treatment of severe NP.

## Methods

Twenty ICU patients with NP were enrolled in the study (all male, 49 ± 7.3 years old); primary reason for ICU stay - intraabdominal infections (60%), mediastinitis (10%), others (30%). Diagnosis of NP was made according to standard clinical and CPIS criteria. Associations of multiresistant Gram-negative bacteria were detected in bronchoalveolar lavage (BAL) of all patients. Eighty percent of bacteria were sensitive to tobramycin. Patients were randomized into two groups - 'IT' (group 1, *n *= 10) + systemic antibiotics (carbapenems, aminoglycosides, protected penicillins); 'no IT' (group 2, *n *= 10), only systemic antibiotics, same as in group 1. Groups were comparable in APACHE II and CPIS scores. IT (Bramitob) was administered 300 mg BID via nebulizer.

## Results

Duration of IT use in group1 was 7.5 ± 2.5 days. There were no statistically reliable differences between groups detected due to the small number of patients enrolled. But it was clinically detected that treatment with IT in group1 was associated with a decrease of SIRS signs and CPIS scores and an increase of oxygenation index in 70% of patients. Positive dynamics in chest X-ray and computed tomography was detected in two patients of group 1 (20%; no dynamics in group 2). The titre of microbes in BAL decreased (100%) and their sensitivity to other groups of antibiotics, which they were previously resistant to, increased (40%) in group 1 patients after IT administration. Efficacy of IT in patients with a registered resistance of microbes to tobramycin can be explained by a high local concentrations of tobramycin in lungs. The mortality in groups was similar (40% and 40%) and not related to a progression of NP. Two patients of group 1 (20%) presented with hearing loss and tinnitus which revealed 3 months after the last IT administration. There were no cases of bronchospasm or renal insufficiency in group 1.

## Conclusion

Administration of IT as an adjunct to systemic antibiotics is efficient and safe in treatment of severe nosocomial pneumonias caused by multiresistant Gram-negative bacteria. Profound randomized clinical trials on IT are required.
